# Relapsing Candida parapsilosis Endocarditis With Septic Embolization: A Case Report

**DOI:** 10.7759/cureus.13159

**Published:** 2021-02-05

**Authors:** Francisco Teixeira da Silva, Francisca S Cardoso, Alexandra Esteves, José Carvalho, Rosana Maia

**Affiliations:** 1 Internal Medicine, Unidade Local de Saúde do Alto Minho (ULSAM), Viana do Castelo, PRT; 2 Critical Care Medicine, Unidade Local de Saúde do Alto Minho (ULSAM), Viana do Castelo, PRT

**Keywords:** invasive fungal disease, echocardiography - heart failure - valvular heart disease, candida endocarditis, valvular endocarditis, candidemia

## Abstract

*Candida* endocarditis is a rare infection that is becoming an emerging and growing health concern, especially among risk groups such as the elderly and the immunosuppressed. It is associated with high morbidity and mortality. Dilemmas about *Candida* endocarditis treatment are still around, particularly about the treatment options and their duration.

We report a case of *Candida parapsilosis* prosthetic valve endocarditis with septic embolisms. An elderly male patient with a biological prosthetic valve presented with fever and constitutional symptoms. Abdominal computed tomography (CT) showed an area suggestive of splenic emboli. Transesophageal echocardiography showed a vegetation attaching to the prosthetic valve. Due to several comorbidities, he was not considered a candidate for surgical treatment. He was treated with antifungal drugs (liposomal amphotericin B and caspofungin) and was discharged with *per os* fluconazole. Later he presented with evidence of lumbar spondylodiscitis due to septic embolization and relapsing fungemia with multidrug-resistant isolates was documented. Unfortunately, the patient outcome was ill-fated and he died in hospital due to sepsis-related to the candidemia and also nosocomial urinary sepsis. Here, we illustrate the complexity of diagnosing and managing fungal endocarditis due to its complications and poor prognosis.

## Introduction

Disseminated fungal infections are rare conditions and fungal endocarditis (FE) is even more unique. FE represents only 2%-4% of all endocarditis [1,2]. The incidence of *Candida* endocarditis has been increasing simultaneously with the increase in the general number of fungal infections. Most common known risk factors for FE include intravenous (IV) drug users, patients with prosthetic heart valves, immunocompromised hosts (namely transplant recipients), cancer patients receiving chemotherapy, prolonged use of a central venous catheter (CVC), human immunodeficiency virus infection, and a previous episode of bacterial endocarditis [1,3]. Recent series confirm that *Candida* endocarditis is becoming a predominantly healthcare-associated infection (87%) [3,4].

FE has a remarkably high number of complications and burden of disease [5]. The mortality rate is between 30% to 50% [6,7]. Contributing factors include the host immune state, often delayed or missed diagnosis, and lack of efficient antifungal agents in the absence of surgery. Also worth noting, FE is associated with significant recurrence rates and relapses have been documented months to years later [1,8,9].

## Case presentation

We present the case of an 81-year-old male with a previous medical history of arterial hypertension, peripheral arterial disease (for which he had received aorto-bifemoral bypass six years before). Three years earlier he had also undergone a biological aortic valve replacement surgery due to aortic stenosis.

A recent medical history included a prolonged hospitalization early that year when he was diagnosed with gastric adenocarcinoma - staging pT1N0M0. He underwent a subtotal gastrectomy with Billroth type II anastomosis. In the postoperative period, he developed a stricture of the gastrojejunostomy. It was managed conservatively and he received long-term parenteral nutrition (PN) through CVC. He scored 0 points in the Eastern Cooperative Oncology Group (ECOG) performance status but needed no systemic therapy.

**Figure 1:**
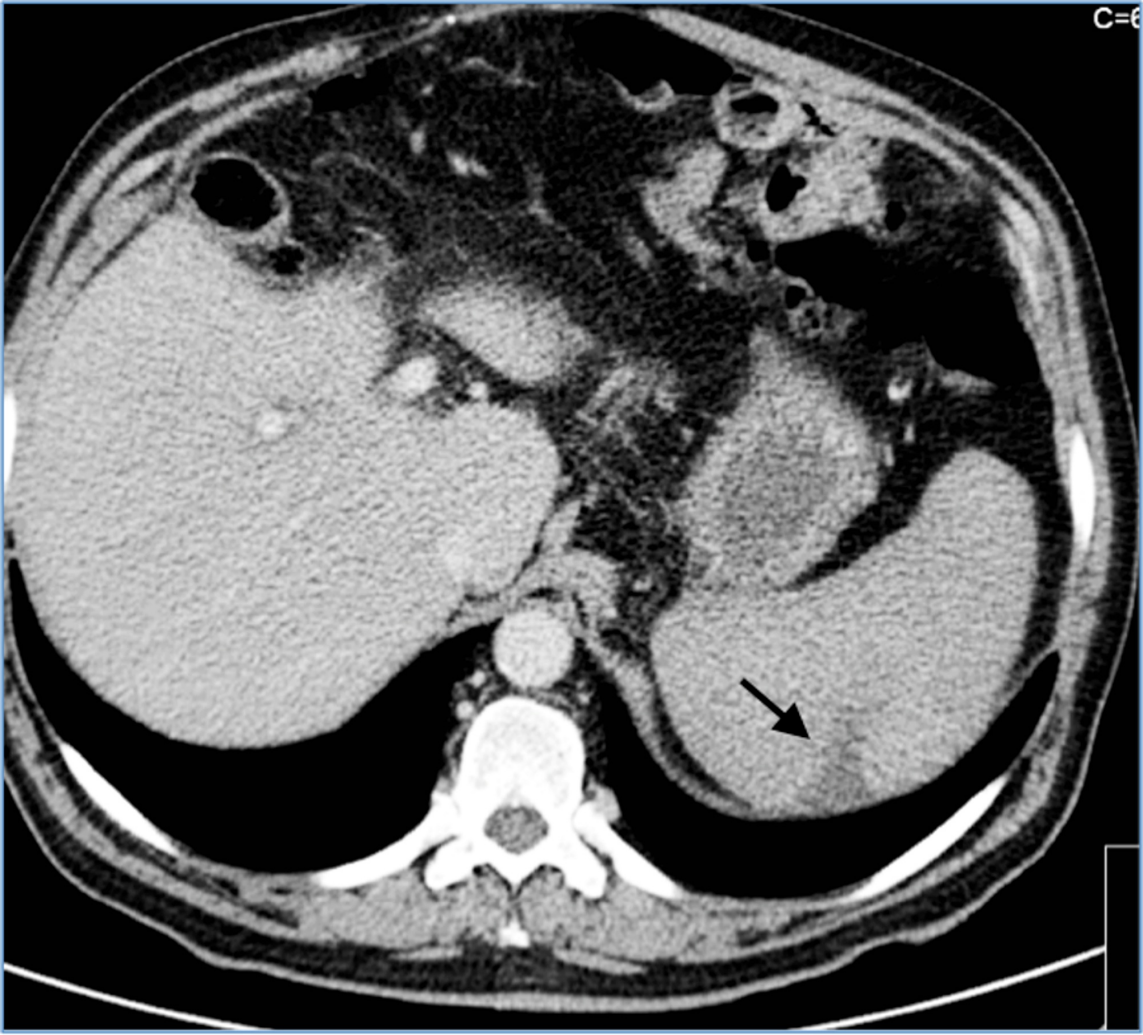
Abdominal CT scan showing an area of spleen infarct (black arrow)

Six months later, he presented to our Emergency Department (ED) complaining of vespertine fever, anorexia, and asthenia. The symptoms had been present for one month. On physical examination, we noted an excellent general appearance, pallor, new-onset grade II/VI aortic systolic murmur, a tender abdomen on palpation, and splenomegaly. The laboratory workup showed microcytic hypochromic anemia (Hg 11.2 g/dL), normal leukocyte count with relative lymphocytosis, altered liver panel, thrombocytopenia, and elevated C reactive protein (CRP) (Table 1). An abdominal CT scan was performed and showed an enlarged spleen with a 32x22mm peripheral, wedge-shaped hypo-enhancing area on its posterior aspect, which was highly suggestive of a splenic infarct (Figure 1). No other signs of peripheral emboli were noted.

**Table 1:** Laboratory workup MCHC: mean corpuscular hemoglobin concentration, MCV: mean corpuscular volume, CRP: C-reactive protein, Hct: hematocrit

Tests	Reference values	Results
Haemoglobin (g/dL)	11.8 - 15.8	11.2
Hct (%)	36 - 46	36
MCV (fL)	80.4 - 96.4	74.8
MCHC (g/dL)	31,7 - 35,7	31.1
Leucocytes (μL)	4.0 - 10.0	5480
Neutrophils	1800–7700	2.049
Lymphocytes	800–4000	2.619
Platelets (10^9/uL)	150 - 400	32
Glucose (mg/dL)	70 - 110	99
Urea (mg/dL)	17 - 43	26
Creatinine (mg/dL)	0.6 - 1.0	0,83
Sodium (mmol/L)	136 - 145	139
Potassium (mmol/L)	3.5 - 5.1	4,4
CRP (mg/dL)	0.01-0.82	7,2
Total / direct bilirubin (mg/dL)	0.3 - 1.2	1.19 / 0.6
Alkaline phosphatase (UI/L)	30 - 120	255
Gamma-glutamyl Transferase (UI/L)	<55	110
Aspartate Transaminase (UI/L)	8 - 35	93
Alanine Transaminase (UI/L)	10 - 45	26

The patient was admitted to the Internal Medicine ward with a presumptive diagnosis of infective endocarditis with splenic emboli. He met three minor clinical criteria from the Modified Duke Criteria for the Clinical Diagnosis of Infective Endocarditis [10] - fever, a predisposing heart condition, and systemic arterial emboli. Empirical antibiotic therapy with vancomycin and gentamicin was started - considering it might be healthcare-associated endocarditis due to recent hospitalization and abdominal surgery. Rifampicin was added three days later, as advocated for prosthetic valve (PV) infective endocarditis (IE) by The Task Force for the Management of Infective Endocarditis of the European Society of Cardiology (ESC) [11].

A transthoracic echocardiogram (TTE) was performed and no changes suggestive of endocarditis were noted. Due to a high suspicion index, a transesophageal echocardiogram (TEE) was performed: it revealed a 7x7mm vegetation attached to the right coronary cusp of the prosthetic aortic valve; no perivalvular abscess, leaks, or prosthetic dysfunction were present (Video 1, 2).

**Video 1:** TEE showing vegetation attached to the right coronary cusp of the prosthetic aortic valve. Upper esophageal probe position - Aortic valve long axis-view. TEE, transesophageal echocardiogram.

**Video 2:** TEE showing vegetation attached to the right coronary cusp of the prosthetic aortic valve. Upper esophageal probe position - Aortic valve short-view. TEE, transesophageal echocardiogram

Blood cultures were negative for bacterial agents and yielded colonies of *Candida parapsilosis*. After this result, the patient was started on antifungal therapy with liposomal amphotericin B (5mg/kg daily), as recommended on current guidelines [8]. He developed a severe adverse anaphylactic reaction and treatment had to be suspended. An alternative regimen with high-dose echinocandin (caspofungin 150mg daily) was initiated, also according to latest recommendations [8]. The antibiotic susceptibility testing (AST) was only available two weeks later: it documented sensitivity to flucytosine, amphotericin B, fluconazole, and voriconazole but resistance to caspofungin. Accordingly, the patient was started on fluconazole therapy (12mg/kg/day).

Surgery was discussed between practitioners from several medical specialties (an *ad hoc* Endocarditis Team) but the patient was not considered for aortic replacement surgery, considering his comorbidities and very high anesthetic risk with a EuroSCORE II of 15.59% and a Society of Thoracic Surgeons (STS) score of 16.76%. A decision was made for lifelong suppressive therapy with fluconazole, as per current guidelines [8,11]. The patient was discharged from hospital once clinically stable and after two sets of negative blood cultures (over 24 hours apart). 

**Figure 2:**
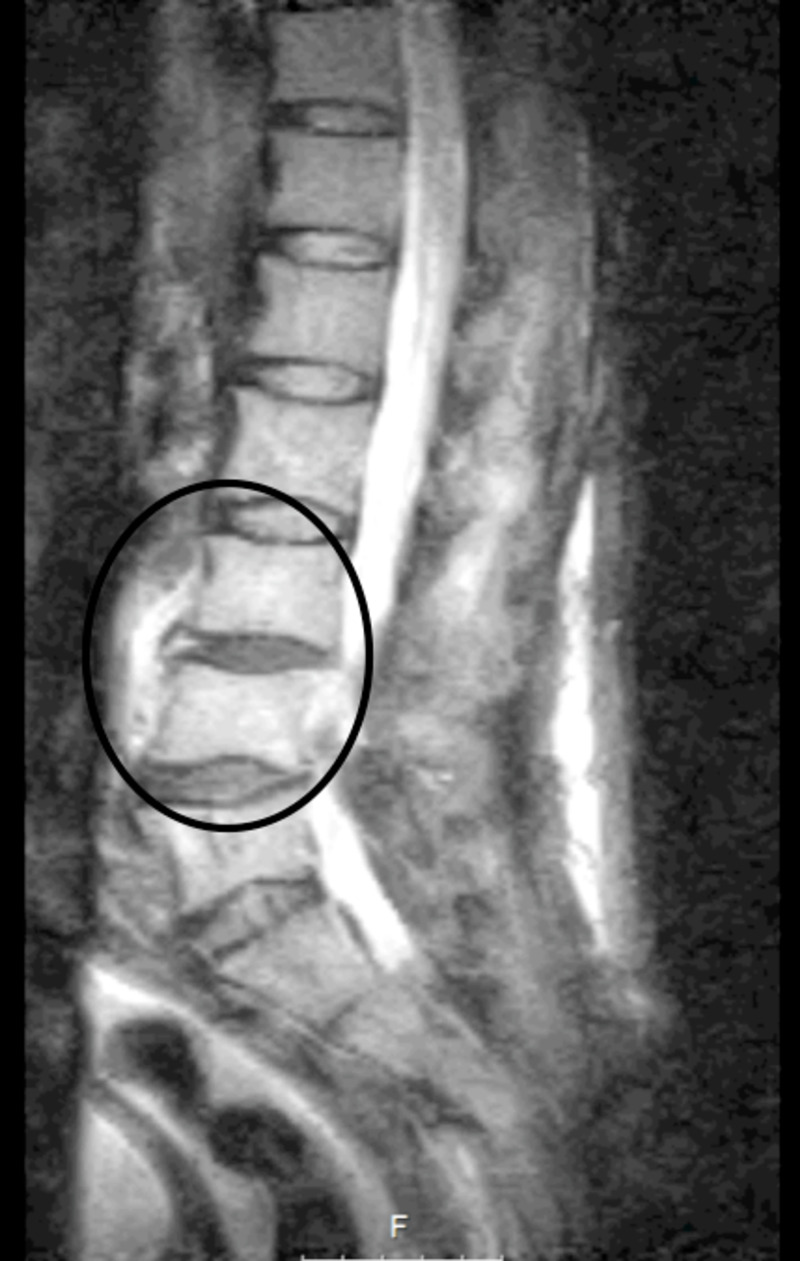
MRI T2 sequences showing L3-L4 spondylodiscitis (black circle). MRI, magnetic resonance imaging

Two months after discharge, he was admitted to our ED complaining of severe lower back pain, fever, and vomiting. By this time, the patient's ECOG performance status had declined significantly from 0 to 3. He underwent lumbar magnetic resonance imaging (MRI), which showed high signal in adjacent endplates and thickening of paravertebral soft tissues (T2 sequences) surrounding the L3-L4 intervertebral disk (Figure 2). These changes were compatible with an indolent manifestation of lumbar spondylodiscitis due to septic embolization. Microbiological relapse was documented. Blood cultures grew the same agent, but *de novo* resistance to fluconazole was reported in AST.

The patient developed urinary sepsis with multiorgan failure due to extended-spectrum beta-lactamases (ESBL) *Klebsiella pneumonia* with documented bacteriemia. Unrelenting clinical deterioration culminated with the patient’s death in-hospital approximately three months after the diagnosis of FE. The final diagnosis was *Candida parapsilosis* endocarditis with septic embolization (splenic infarct and spondylodiscitis).

## Discussion

FE is an uncommon but dangerous and devastating infection. *Candida* species are the most frequent agents involved in FE: *C. albicans* cause approximately 25% of cases, non-*Candida albicans* Candida (NCAC) strains cause ~25% and other fungi such as *Aspergillus* are responsible for the remaining percentage [3].

*C. parapsilosis* was originally considered non-pathological until 1940 when it was identified as the causative agent of an FE that caused the death of an IV drug user [12]. Nowadays, *C. parapsilosis* is the second most common strain to be isolated from blood culture and is associated with nosocomial infections. It is mostly related to vascular devices [3], due to biofilm production on foreign bodies and indwelling catheters. [13]. The biofilm structures form pseudo-hyphae (called "giant cells") that are morphologically distinct from those of *C. albicans* [5,13]. They also show increased appetence and growing capacity on CVC used with parenteral hyper-alimentation solutions [5].

Predisposing risk factors for *C. parapsilosis* include the prosthetic heart valves or devices (57.4%), IV drug use (20%), PN (6.9%), immunosuppression (6.4%), treatment with broad-spectrum antibiotics (5.6%) and previous valvular disease (4.8%) [13]. When compared to *C. albicans*, *C. parapsilosis* FE has a more frequent history of valvular disease and prior parenteral nutrition [13]. *C. parapsilosis* has also been linked to indwelling CVCs, cardiac devices, transcatheter aortic valve replacement (TAVR), long-term glucocorticoid therapy and transplant recipients [5,6,8]. All these risk factors seem to have a cumulative behaviour [6,14].

Endocarditis presenting symptoms and signs include fever (in 90% of patients), which is usually protracted (>two weeks) and accompanied by chills, sweating, and malaise [15]. Dyspnea, heart failure (HF), and peripheral embolisms might be present [16]. New-onset murmur or changes in a previously known murmur (in 75% of patients) should also be considered [6]. Presentation can be acute or subacute, with nonspecific symptoms over weeks or months. Microembolic or immunologic phenomena (as splinter hemorrhages, conjunctival hemorrhages, Osler nodes, Janeway lesions, and Roth spots) can be seen in 5-10% of patients [15].

FE signs and symptoms are generally comparable to those of bacterial etiology, but FE has some distinctive features [8,13]. FE is characterized by (i) larger (‘bulky’) vegetations, responsible for an increased risk of drastic embolic events (such as massive stroke [6] or member ischaemia [17] ) and valvular destruction or chordae rupture leading to acute mitral insufficiency; (ii) more ophthalmological complications, with typical findings on fundoscopy; and (iii) specific dermatological conditions unique to fungal pathogens - macronodules or maculopapules in candidaemia and black hemorrhagic lesions in *Aspergillus* endocarditis have been reported [18].

One should suspect FE in cases of recurrent fever in patients with a past history of fungaemia [9], especially in patients with blood culture-negative IE (BCNIE) - i.e. IE in which no causative microorganism can be grown using the usual blood culture methods [11]. Despite vegetations seen on echocardiography, blood cultures are negative in over 50% of cases [6].

Diagnosis is based mainly on two aspects: microbiologic tests and echocardiogram (other imaging modalities are also available). A positive blood culture result is highly desirable. Susceptibility testing and determination of fungicidal minimum inhibitory concentrations (MICs) are mandatory [3]. However, the sensitivity for the diagnosis of FE has been estimated at 50-75% or lower [18]. Explanted valves and tissue should also be cultured for fungi/bacteria.

New testing alternatives are also emerging. The mannan antigen and antibody tests for candidaemia detect circulating *Candida* antigens, antibodies, or other metabolites and have a combined sensitivity and specificity of 83% and 86% respectively for diagnosing fungaemia. This represents an estimated accuracy of 50-70% [18]. Polymerase chain reaction (PCR) molecular methods are also available in blood or in explanted valves and are 3-fold more sensitive than Gram staining and culture [6,15]. However, there is currently no evidence to support the use of these tests in diagnosis of FE and treatment decisions should not be made based on these results alone [3]. 

TEE has higher sensitivity compared to TTE in detecting vegetations (95% vs. 60%) [18]. Other organ involvement must also be excluded (due the high embolic rate of events) and fundoscopy should be performed as well as an active search for a thrombus elsewhere [7].

Treatment options contemplate a multimodal approach including combined antifungal agents and surgery for the successful management of FE. Early as possible and aggressive surgical treatment is recommended (class I indication, level of evidence B) due to the high mortality and morbidity among patients with medical treatment alone [6,7].

Native or prosthetic valve FE, is considered a standalone indication for surgery by most society guidelines - including the American College of Cardiology (ACC), the American Heart Association (AHA), and the Infectious Diseases Society of America (IDSA) [13,18]. Surgery should be performed as soon as possible, ideally in the first week [7]. Paradoxically, some studies did not find differences in mortality between those undergoing surgical therapy and those receiving only medical therapy [4]. Rare cases of successful treatments with medical therapy alone have been described [19]. Due to lack of randomized studies, there is no consensus on the optimal medical treatment nor its duration [7,20].

Therapeutic recommendations for initial therapy of native valve endocarditis include lipid formulation amphotericin B, 3-5 mg/kg daily, with or without flucytosine, 25 mg/kg four times daily, *OR* a high-dose echinocandin (caspofungin 150 mg daily, micafungin 100-150 mg daily, or anidulafungin 100-200 mg daily) [7,8,18]. Azoles are only fungistatic in yeasts and therefore cannot be used as primary treatment of *Candida* endocarditis [18].

Step-down therapy to fluconazole - 400-800 mg (6-12 mg/kg) daily - is recommended for patients who have susceptible *Candida* isolates, clinical stability, and evidence fungaemia clearance [8]. A minimum of 14 days after the end of candidaemia (determined by one blood culture per day until negativity) is recommended and switching to oral treatment after 10 days of intravenous therapy is proved safe in clinically stable patients with susceptible species [7]. Some authors suggest courses of six weeks or longer in patients with perivalvular abscesses and other complications [8]. When valve replacement is NOT an option after the initial treatment long-term, suppression with fluconazole - 400-800 mg (6-12 mg/kg) daily - is a reasonable option if the isolate is susceptible [6-8,11]. The same antifungal regimens are recommended for prosthetic valve endocarditis. Some authors also advocate chronic suppressive antifungal therapy with fluconazole - 400-800 mg (6-12 mg/kg) daily - to prevent recurrence [8].

Echinocandin and azole resistance in *Candida *spp. are becoming a particular concern [13]. Persistent fungaemia after one week of treatment should raise suspicion of resistance and susceptibility should be tested, as resistance may emerge on therapy [3]. *C. parapsilosis* is usually susceptible to amphotericin B, flucytosine, and azoles. Echinocandin-resistant strains (as in this case report) are infrequent and have only been described in case reports [8,20]. One explanation is that *C. parapsilosis* demonstrates intrinsically higher MICs to the echinocandins than other *Candida* spp. However there have been no clinical studies proving fluconazole superiority over the echinocandins for *C. parapsilosis *infections [8].

Despite progress in antifungal therapy and surgical techniques, prognosis is poor and one-year mortality rate remains as high as 50% [7,8,11]. Baseline characteristics associated with high mortality are: older age, previous HF, and nosocomial acquisition of FE [4,9]. Higher mortality was also associated with clinical development of new-onset congestive HF and refractory candidemia [4]. Relapsing FE is a complication seen in as many as 30 to 40%.

Treatment failure causes include relapses due to *Candida* species ability to form biofilms, which reduce action of anti-fungal agents [5]. Surgical technical difficulties also play a role: homograft appears to be the most appropriate choice by allowing complete debridement of infected tissue with low risk of valve dehiscence and better antibiotic penetration [19]. Other challenges to consider in treating *Candida* endocarditis include geographic variations, virulence, and reduced susceptibility to antifungal medications [5].

## Conclusions

FE is rare, but it carries a high mortality and incidence has been increasing over the last decades. *Candida* endocarditis clinical spectrum ranges from native or prosthetic valve endocarditis to infection of cardiac devices. Treatment options include combined antifungal agents and surgery.

This case report reminds us of the importance of raising awareness for fungal disease. Management must be made on a case-by-case personalized basis, considering multiple variables such as antifungal agents, surgical options as well as disease severity, prognosis, and patient’s status.

New evidence and recommendations will surely provide more guidelines to improve clinical medicine and contribute to more successful management of FE. Areas of intervention should include policies aiming at reducing FE in select populations (e.g. needle exchange programs, prophylactic usage of fluconazole in selected patients), innovative diagnostic tests, and expert or society consensus on the definition of clinical or microbiological criteria to guide diagnosis and treatment options for these patients.
